# Tryptophan Ameliorates Barrier Integrity and Alleviates the Inflammatory Response to Enterotoxigenic *Escherichia coli* K88 Through the CaSR/Rac1/PLC-γ1 Signaling Pathway in Porcine Intestinal Epithelial Cells

**DOI:** 10.3389/fimmu.2021.748497

**Published:** 2021-10-21

**Authors:** Guangmang Liu, Ke Gu, Fang Wang, Gang Jia, Hua Zhao, Xiaoling Chen, Caimei Wu, Ruinan Zhang, Gang Tian, Jingyi Cai, Jiayong Tang, Jing Wang

**Affiliations:** ^1^Institute of Animal Nutrition, Sichuan Agricultural University, Chengdu, China; ^2^Key Laboratory for Animal Disease-Resistance Nutrition, Ministry of Education, Chengdu, China; ^3^Key Laboratory of Animal Disease-Resistant Nutrition and Feed, Ministry of Agriculture and Rural Affairs, Chengdu, China; ^4^Maize Research Institute, Sichuan Agricultural University, Chengdu, China

**Keywords:** tryptophan, inflammatory response, intestinal tight junctions, intestinal permeability, CaSR/Rac1/PLC-γ1 signaling pathway

## Abstract

**Background:**

Impaired intestinal barrier integrity plays a crucial role in the development of many diseases such as obesity, inflammatory bowel disease, and type 2 diabetes. Thus, protecting the intestinal barrier from pathological disruption is of great significance. Tryptophan can increase gut barrier integrity, enhance intestinal absorption, and decrease intestinal inflammation. However, the mechanism of tryptophan in decreasing intestinal barrier damage and inflammatory response remains largely unknown. The objective of this study was to test the hypothesis that tryptophan can enhance intestinal epithelial barrier integrity and decrease inflammatory response mediated by the calcium-sensing receptor (CaSR)/Ras-related C3 botulinum toxin substrate 1 (Rac1)/phospholipase Cγ1 (PLC-γ1) signaling pathway.

**Methods:**

IPEC-J2 cells were treated with or without enterotoxigenic *Escherichia coli* (ETEC) K88 in the absence or presence of tryptophan, CaSR inhibitor (NPS-2143), wild-type CaSR overexpression (pcDNA3.1-CaSR-WT), Rac1-siRNA, and PLC-γ1-siRNA.

**Results:**

The results showed that ETEC K88 decreased the protein concentration of occludin, zonula occludens-1 (ZO-1), claudin-1, CaSR, total Rac1, Rho family member 1 of porcine GTP-binding protein (GTP-rac1), phosphorylated phospholipase Cγ1 (p-PLC-γ1), and inositol triphosphate (IP_3_); suppressed the transepithelial electrical resistance (TEER); and enhanced the permeability of FITC-dextran compared with the control group. Compared with the control group, 0.7 mM tryptophan increased the protein concentration of CaSR, total Rac1, GTP-rac1, p-PLC-γ1, ZO-1, claudin-1, occludin, and IP_3_; elevated the TEER; and decreased the permeability of FITC-dextran and contents of interleukin-8 (IL-8) and TNF-α. However, 0.7 mM tryptophan+ETEC K88 reversed the effects induced by 0.7 mM tryptophan alone. Rac1-siRNA+tryptophan+ETEC K88 or PLC-γ1-siRNA+tryptophan+ETEC K88 reduced the TEER, increased the permeability of FITC-dextran, and improved the contents of IL-8 and TNF-α compared with tryptophan+ETEC K88. NPS2143+tryptophan+ETEC K88 decreased the TEER and the protein concentration of CaSR, total Rac1, GTP-rac1, p-PLC-γ1, ZO-1, claudin-1, occludin, and IP_3_; increased the permeability of FITC-dextran; and improved the contents of IL-8 and TNF-α compared with tryptophan+ETEC K88. pcDNA3.1-CaSR-WT+Rac1-siRNA+ETEC K88 and pcDNA3.1-CaSR-WT+PLC-γ1-siRNA+ETEC K88 decreased the TEER and enhanced the permeability in porcine intestine epithelial cells compared with pcDNA3.1-CaSR-WT+ETEC K88.

**Conclusion:**

Tryptophan can improve intestinal epithelial barrier integrity and decrease inflammatory response through the CaSR/Rac1/PLC-γ1 signaling pathway.

## Introduction

Enterotoxigenic *Escherichia coli* (ETEC) invasion causes intestinal damage and diarrhea in children and young animals. Piglets are inclined to ETEC K88-induced diarrhea because the bacteria can produce enterotoxins. These substances destroy the intestinal mucosal layer and tight junction (TJ) structure, which increases the permeability of the intestine, ultimately causing intestinal inflammation (1–4). ETEC K88-induced diarrhea can lead to great economic loss in the pig industry (1–4). ETEC K88 can modulate epithelium barrier function by inducing cellular signals such as the toll-like receptors (TLR) and p38/mitogen-activated protein kinase (MAPK) signaling pathway in intestinal epithelial cells (5, 6). Pathogenic ETEC K88 can activate innate immunity and induce inflammatory reactions through the nuclear factor kappa-B (NF-κB), TLR4, and MAPK signaling pathways (7–9).

Tryptophan, one of the functional amino acids, has been reported to improve the growth, decrease stress-induced injury, improve appetite and mitochondrial function, enhance antioxidant status, increase immunity, enhance the diversity of the intestinal microbiome, change anabolism, and improve intestinal wound restitution in animals (10–16). In particular, tryptophan plays a vital role in protecting intestinal integrity by regulating the expression of TJ proteins (17–19). The transepithelial electrical resistance (TEER) and permeability reflect the integrity and function of the intestinal epithelium layer and are utilized to evaluate pathogenic microorganisms’ challenges (18). Nevertheless, the effects of tryptophan supplementation on intestinal TEER and permeability in ETEC K88-induced intestinal epithelial cells have not been investigated. Lack of tryptophan can change the gut microbial ecosystem and lead to intestinal inflammation (20). Additionally, tryptophan supplementation reduces the mRNA levels of proinflammatory cytokines interleukin-8 (IL-8) and IL-1β in the gut (21). However, the exact molecular mechanisms by which tryptophan contributes to intestinal barrier integrity and inflammation response of intestinal epithelial cells remain unknown.

The calcium-sensing receptor (CaSR) plays critical roles in the regulation of intestinal inflammation, intestinal epithelium restitution, and intestinal TJ protein expression (21–24). Tryptophan induces the activation of CaSR, which decreases the mRNA levels of proinflammatory cytokines IL-8 and IL-1β in piglets, suggesting that the CaSR signaling pathway may be involved in intestinal inflammatory response (21, 25). A research in mice reported that the suppression of CaSR could improve FITC-conjugated dextran and decrease the TEER in the intestine (26). CaSR overexpression can enhance IPEC-J2 cell migration (24). CaSR stimulation increased zonula occludens-1 (ZO-1) and F-actin-binding protein interaction in Madin–Darby canine kidney (MDCK) cells (27). However, whether tryptophan influences intestinal barrier permeability and TJ proteins through CaSR signaling remains unknown. The activation of CaSR results in the activation of Ras-related C3 botulinum toxin substrate 1 (Rac1) and phosphorylation of phospholipase Cγ1 (PLC-γ1), which are involved in inflammatory response and intestinal epithelial cell migration (24, 28–30).

Rac is a key target that modulates the permeability of paracellular pathways (31). Rac1 is required for TJ barrier during epithelial junction assembly and intestinal inflammatory response (28). In mouse studies, CaSR inhibition prevents the protein expression of Rac/Cdc42 and claudin-1, claudin-4, and claudin-5 (32). Compared with pcDNA3.1(+)-pCaSR, pcDNA3.1(+)-pCaSR+Rac1-siRNA significantly decreased cell migration (24). However, whether the CaSR/Rac1 signaling pathway is involved in tryptophan-influenced inflammatory response, intestinal TJ expression, TEER, and permeability of intestinal cells remains unknown. PLC-γ1 is involved in regulating intestinal inflammation, epithelial TJ, and permeability (33, 34). Compared with pcDNA3.1(+)-pCaSR, pcDNA3.1(+)-pCaSR+PLC-γ1-siRNA significantly decreased cell migration (24). However, whether PLC-γ1 is involved in the mechanism of tryptophan in modulating intestinal inflammation, TJ, and permeability *via* CaSR signaling in intestinal cells is unclear. Our previous reports showed that tryptophan increased intestinal epithelial cell migration though the CaSR/Rac1/PLC-γ1 signaling pathway (24). However, whether tryptophan modulates intestinal inflammation, TJ, and permeability though the CaSR/Rac1/PLC-γ1 signaling pathway after challenge with ETEC K88 remains unknown. This study aimed to test the hypothesis that tryptophan can enhance intestinal epithelial barrier integrity and reduce inflammatory response mediated by the CaSR/Rac1/PLC-γ1 signaling pathway.

## Materials and Methods

### Materials

Tryptophan (≥99%, #T8941) and FITC-dextran 4kDa (FD4, #BCCC6414) were purchased from the Sigma-Aldrich (MO, USA). Dulbecco’s Modified Eagle Medium : Nutrient Mixture F-12 (DMEM/F12, #C11330500BT), fetal bovine serum (FBS, #10099141C), penicillin/streptomycin (S/P, #15140122), and trypsin (#25200-056) were purchased from Gibco (USA). CaSR inhibitor NPS2143 (#S2633) was purchased from Selleck (Houston, USA). LipofectAMINE 3000 (#2304049) was purchased from Invitrogen (Carlbad, CA, USA). *Escherichia coli* K88 (ETEC: serotype O149:K91: K88ac) was purchased from the China Institute of Veterinary Drugs Control (Beijing, China). IPEC-J2 cells was preserved in our lab.

### Bacterial Strains and Culture

*Escherichia coli* K88 was cultured in 10 mL of sterilized (121°C, 0.11 MPa for 20 min) Luria–Bertani (LB) medium (peptone, 1 g; NaCl, 1 g; yeast extract, 0.5 g; double-distilled water, 100 mL) overnight under shaking at a speed of 200 rpm at 37°C. About 100 μL of the bacterial solution was resuspended with 5 mL of sterilized LB medium and shaken at a speed of 250 rpm at 37°C for 2 h (35). Bacterial concentrations were determined from standard curves generated by multiplicity of infection. IPEC-J2 cells were washed with sterile phosphate buffered saline (PBS), which was replaced with 2% FBS medium without antibiotics. Then, the cells were incubated with ETEC K88 (1 × 10^8^ CFU/mL) for 2 h. The selection of this serotype of ETEC K88 was based on previous study (35).

### Cell Culture

IPEC-J2 cells were cultured in DMEM/F12 supplemented with 10% FBS and 100 IU/mL penicillin/100 μg/mL streptomycin at 37°C with 5% CO_2_ atmosphere.

### Small Interfering RNA (siRNA) and Plasmid Transfection

The siRNAs directed specifically against PLC-γ1 and Rac1 were designed on the basis of the sequence of PLC-γ1 (GenBank accession no. NM_021078391.1) and Rac1 (GenBank accession no. NM_001243585.1). The sequences of siRNAs [PLC-γ1-siRNA, Rac1-siRNA, and negative control siRNA (NC-siRNA)] are listed in **Table 1**. NC-siRNA was used as the control. siRNAs were synthesized and obtained from Gene Pharma (Shanghai, China). siRNAs were dissolved in DPEC water to obtain the final concentration of 50 nM. CaSR overexpression plasmids (pcDNA3.1-CaSR) and pcDNA3.1+ were synthesized and purchased from Youbio Biotechnology Co., Ltd. (Changsha, China). Lipofectamine 3000 was used to transfect IPEC-J2 cells according to the manufacturer’s instructions.

**Table 1:** Sequence of siRNA.

Gene names		Sequence
PLCγ1-siRNA	sense	5′-CCAGAAGUGCGACACCAUUTT-3′
	antisense	5’-GCCCTCTGGGTATGGCTTTC-3’
Rac1-siRNA	sense	5’- CCAAGGAUCUGAAGAACAUTT-3’
	antisense	5’- AUGUUCUUCAGAUCCUUGGTT-3’
NC-siRNA	sense	5’-UUCUCCGAACGUGUCACGUT-3’
	antisense	5’-ACGUGACACGUUCGGAGAATT-3’

### Cell Treatment

The IPEC-J2 cells (1 × 10^6^ cells/mL) were seeded in 6-well Costar plates (Corning, New York, USA) and incubated with 10% FBS medium. The cells were treated as follows: (1) When the cells reached approximately 70% confluence, they were incubated in FBS-free medium for 6 h. Then, they were treated with 0.3 or 0.7 mM tryptophan in 2% FBS medium for 48 h, followed by ETEC K88 for 2 h in 2% FBS medium without antibiotics. (2) The cells were pre-incubated with NPS2143 for 1 h, followed by 0.7 mM tryptophan for 48 h, and then they were treated with ETEC K88 for 2 h in 2% FBS medium without antibiotics. (3) The IPEC-J2 cells were transfected with 1.25 μg/mL of pcDNA3.1-CaSR and 50 nM NC-siRNA, Rac1-siRNA, or PLC-γ1-siRNA for 48 h in a 10% FBS medium, and then treated with ETEC K88 for 2 h in 2% FBS medium without antibiotics. (4) The cells were pre-incubated with 50 nM NC-siRNA, Rac1-siRNA, or PLC-γ1-siRNA for 12 h, followed by 0.7 mM tryptophan for 48 h, and then they were treated with ETEC K88 for 2 h in 2% FBS medium without antibiotics.

### TEER and Permeability Assay

The TEER and FD4 flux of porcine intestinal epithelial cells were detected according to the method of a previous study (36). Briefly, the IPEC-J2 cells (5 × 10^5^/mL) were seeded in 12-well transwell insert (1.12 cm^2^, 0.4 μm) with collagen-coated PTFE membrane (Corning Inc., NY, USA) with 0.5 mL of 10% FBS medium in transwell inserts and 1.5 mL of 10% FBS medium in the plate well. The medium was replaced daily. When the TEER values reached a plateau, the IPEC-J2 cells were considered to form a monolayer. Then, cells were washed with PBS and treated with different reagents. IPEC-J2 cells in each transwell insert membrane were incubated with different reagents at 37°C for the indicated time and treated with 10 μL of FITC-dextran 4 kDa (10 mg/mL) for 2 h. About 200 μL of the basal medium was utilized for fluorescence analysis in a microplate fluorescence reader (emission, 528 nm; excitation, 485 nm, SpectraMax M2, Molecular Devices, China). The concentrations of FITC-dextran were determined *via* standard curves generated using serial dilution of FITC-dextran.

### Real-Time PCR

The PCR experimental procedure was carried out as previously described (37). Briefly, total RNA from IPEC-J2 cells was extracted by TRIzol reagents (TaKaRa, Chengdu, China). One microliter of total RNA was reverse transcribed to cDNA using the PrimeScript RT reagent Kit (TaKaRa, Chengdu, China) with gDNA Eraser (TaKaRa, Chengdu, China). Samples were run on a real-time PCR system (ABI 7900HT, Applied Biosystems) using SYBR Premix Ex Taq II (TaKaRa, Dalian, China), and the total volume of the system was 10 μL. Samples were thermocycled using the program (41 cycles of 95°C for 10 s, 58°C for 35 s), followed by a melting curve program (65°C for 5 s, 95°C for 15 s), and all PCR reactions were run in triplicate. The gene primers used are listed in **Table S1**. The relative mRNA expression of Rac1, PLC-γ1, and CaSR was calculated using the 2^−ΔΔCt^ method.

### Enzyme-Linked Immunosorbent Assay (ELISA)

ELISA was performed as previously described (37). Briefly, cells were dissolved in RIPA buffer containing 1 mM phenylmethylsulfonyl fluoride (PMSF), and then were sonicated and centrifuged at 4°C. The protein concentration of occludin, zonula occludens 1 (ZO-1), claudin-1, CaSR, total Rac1, Rho family member 1 of porcine GTP binding protein (GTP-rac1), phosphorylated phospholipase Cγ1 (p-PLC-γ1), inositol triphosphate (IP_3_), IL-8 and tumor necrosis factor-alpha (TNF-α) were determined using ELISA kit (Mlbio, Shanghai, China).

### Statistical Analysis

All data was analyzed by one-way analysis of variance (ANOVA) followed by Duncan’s multiple range test using SPSS 21.0 software (SPSS Inc., Chicago, IL, USA). The homogeneity of variances was evaluated by Levene’s test. All results were represented as mean ± standard error of mean (SEM). The significance of differences among treatments were identified at *P-*value <0.05.

## Results

### Tryptophan Improved TEER and Decreased Permeability in Porcine Intestinal Epithelial Cells Challenged With ETEC K88

**Figure 1:**
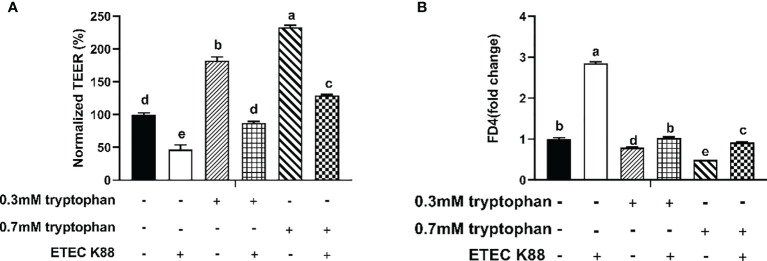
Effect of ETEC K88 and tryptophan (0.3 and 0.7 mM) on the transepithelial electrical resistance and permeability in IPEC-J2 cells. **(A)** Effect of ETEC K88 and tryptophan on the transepithelial electrical resistance value. **(B)** Effect of ETEC K88 and tryptophan on the permeability of FITC-dextran. IPEC-J2 cells were cultured in fetal bovine serum-free medium for 6 h The serum-starved IPEC-J2 cells were pre-treated or not with tryptophan (0.3, 0.7 mM) for 48 h, before challenging or not with ETEC K88 (K88) for 2 h (2% fetal bovine serum DMEM/F12 medium without antibiotic, 1×10^8^ CFU/mL). The TEER value and the permeability of all treatments were normalized to control. Data values are indicated as mean ± SEM (n = 3). Values with different letters indicate significant difference (P < 0.05).

The IPEC-J2 cell monolayer was investigated for epithelial barrier function in response to ETEC K88 infection in the absence or presence of different doses of tryptophan (0.3 and 0.7 mM). Compared with the control group, IPEC-J2 cells treated with ETEC K88 alone showed a spontaneous decrease in TEER value and a significant increase in permeability of FITC-dextran (*P* < 0.05, **Figures 1A, B****)**. Pretreatment with tryptophan (0.3 and 0.7 mM) reversed the ETEC K88-induced reduction of TER value (compared with 0.3 and 0.7 mM tryptophan-treated cells) (*P* < 0.05, **Figures 1A, B****)**. Moreover, treatment with 0.3 and 0.7 mM tryptophan significantly increased the TEER and significantly decreased the permeability of FITC-dextran in IPEC-J2 cell monolayers after 48 h (compared with untreated cells) (*P* < 0.05, **Figures 1A, B****)**. The best protective effect of tryptophan on TEER and permeability was obtained at 0.7 mM concentration. Therefore, we used 0.7 mM tryptophan in subsequent research.

### Rac1/PLC-γ1 Signaling Pathway Contributes to Tryptophan-Induced Upregulation of TEER and Downregulation of Permeability and Inflammatory Response in Porcine Intestinal Epithelial Cells

**Figure 2:**
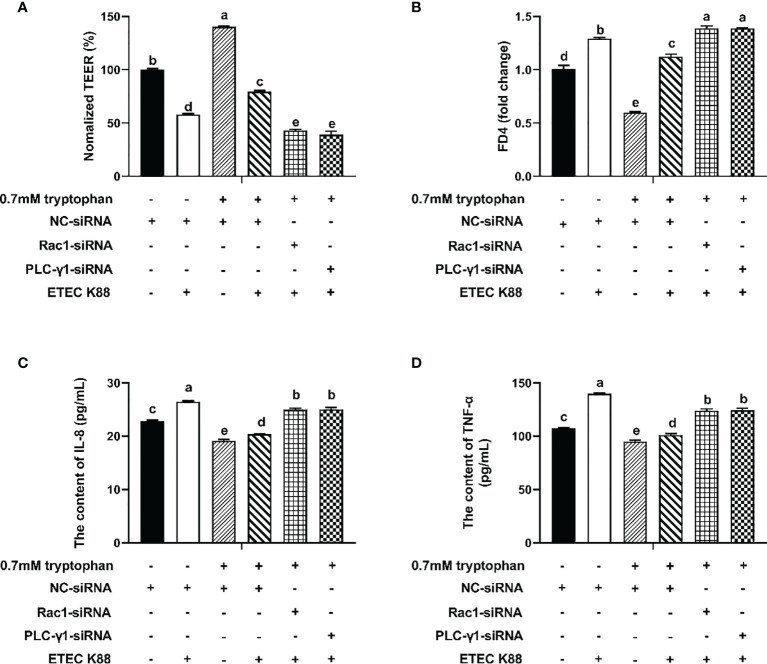
Rac1-siRNA, PLC-γ1-siRNA attenuated the effect of tryptophan (0.7mM) on transepithelial electrical resistance, permeability, and proinflammation cytokines (IL-8 and TNF-α) in ETEC K88-stimulated IPEC-J2 cells. About 70% confluent, IPEC-J2 cells were cultured in fetal bovine serum-free medium for 6 h and then transfected with 50 nM of NC-siRNA, Rac1-siRNA or PLCγ1-siRNA for 24 h, followed by treatment with tryptophan (0.7mM) for 48 h, and then treatment with ETEC K88 for 2h (2% FBS-medium without antibiotic, 1×10^8^ CFU/mL). **(A)** Effect of ETEC K88 and Rac1-siRNA, PLC-γ1-siRNA on the transepithelial electrical resistance value. **(B)** Effect of ETEC K88 and Rac1-siRNA, PLC-γ1-siRNA on the permeability of FITC-dextran. **(C)** Effect of ETEC K88 and Rac1-siRNA, PLC-γ1-siRNA on the contents of IL-8. **(D)** Effect of ETEC K88 and Rac1-siRNA, PLC-γ1-siRNA on the contents of TNF-α. NC-siRNA was added to control, tryptophan and ETEC K88 groups. The TEER value and the permeability of all treatments were normalized to control. Data values are indicated as mean ± SEM (n = 3). Values with different letters indicate significant difference (P < 0.05).

To explore the molecular mechanism by which tryptophan regulates intestinal barrier integrity, IPEC-J2 cells were transfected with Rac1-siRNA or PLC-γ1-siRNA for 24 h before the addition of tryptophan (0.7 mM). Then, the cells were challenged with ETEC K88 for 2 h. The results showed that Rac1-siRNA and PLC-γ1-siRNA significantly decreased Rac1 and PLC-γ1 mRNA expression, respectively (*P* < 0.05, **Figures S1A, B**). Treatment with 0.7 mM tryptophan significantly increased the TEER and significantly decreased the permeability of FITC-dextran and contents of IL-8 and TNF-α in IPEC-J2 cell monolayers compared with untreated cells (*P* < 0.05, **Figures 2A–D**). Compared with the control group, ETEC K88 suppressed the TEER, enhanced the permeability of FITC-dextran, and improved the contents of IL-8 and TNF-α (*P* < 0.05, **Figures 2A–D**). Compared with cells treated with 0.7 mM tryptophan alone, cells treated with 0.7 mM tryptophan + ETEC K88 showed decreased TEER, increased permeability of FITC-dextran, and enhanced contents of IL-8 and TNF-α (*P* < 0.05, **Figures 2A–D**). In addition, compared with treatment with 0.7 mM tryptophan + ETEC K88, Rac1-siRNA or PLC-γ1-siRNA inhibited the tryptophan-induced upregulation of TEER and downregulation of FITC-dextran permeability and IL-8 and TNF-α contents in IPEC-J2 cells challenged with ETEC K88 (*P* < 0.05, **Figures 2A–D**). Taken together, the results suggest that the regulation of TEER, permeability, and inflammatory response by tryptophan is dependent on the Rac1/PLC-γ1 signaling pathway.

### Inhibition of CaSR by NPS2143 Disrupts the Effect of Tryptophan on TJ, Inflammatory Response, TEER, and Permeability in ETEC K88-Challenged IPEC-J2 Cells

**Figure 3:**
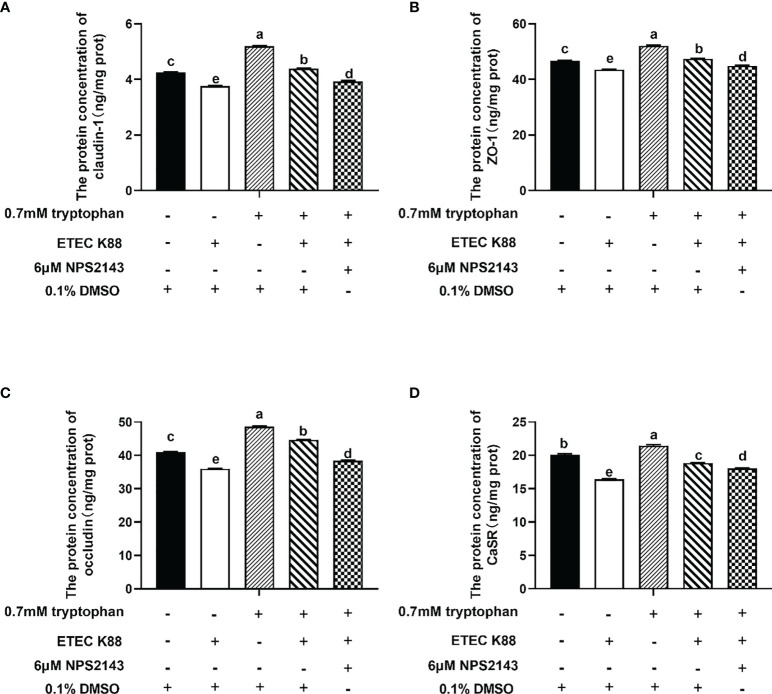
NPS2143 attenuated the effect of tryptophan (0.7mM) on the protein concentration of occludin, ZO-1, claudin-1 and CaSR in ETEC K88-stimulated IPEC-J2 cells. About 70% confluent, IPEC-J2 cells were cultured in fetal bovine serum-free medium for 6 h, and pre-treated with 6μM NSP 2143 or 0.1% of DMSO and for 1 hour, followed by treatment with 0.7 mM tryptophan for 48 h, and then challenged or not with ETEC K88 for 2 h (2% fetal bovine serum-medium without antibiotic, 1×10^8^ CFU/mL). **(A)** CaSR inhibitor NPS2143 attenuated the effect of tryptophan on the protein concentration of claudin-1 in ETEC K88-stimulated IPEC-J2 cells. **(B)** NPS2143 attenuated the effect of tryptophan on the protein concentration of ZO-1 in ETEC K88-stimulated IPEC-J2 cells. **(C)** NPS2143 attenuated the effect of tryptophan on the protein concentration of occludin in ETEC K88-stimulated IPEC-J2 cells. **(D)** NPS2143 attenuated the effect of tryptophan on the protein concentration of CaSR in ETEC K88-stimulated IPEC-J2 cells. Data values are expressed as mean ± SEM from four independent experiments (n = 4). Values with different letters indicate significant difference (P < 0.05).

**Figure 4:**
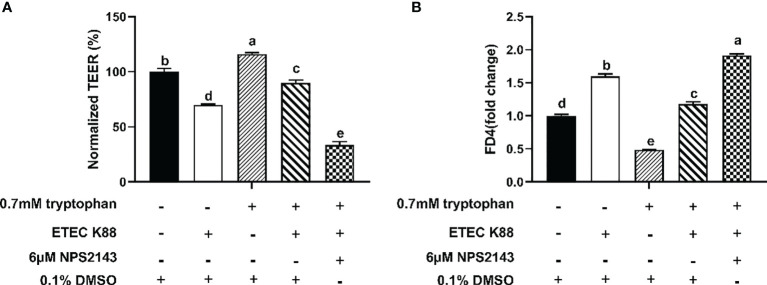
Effect of NPS2143 inhibiting the tryptophan (0.7mM) on the transepithelial electrical resistance and permeability in ETEC K88-stimulated IPEC-J2 cells. The cells were seeded on (5×10^5^/ml) collagen-coated 12-well transwell insert, and after reaching confluence, IPEC-J2 cells were cultured in fetal bovine serum-free medium for 6 h, and pre-treated with 6μM NSP 2143 or 0.1% of DMSO and for 1 hour, followed by treatment with 0.7 mM tryptophan for 48 h, and then challenged or not with ETEC K88 for 2 h (2% fetal bovine serum-medium without antibiotic, 1×10^8^ CFU/mL). **(A)** Effect of NPS2143 and tryptophan on the transepithelial electrical resistance value after stimulation with ETEC K88. **(B)** Effect of NPS2143 and tryptophan on the permeability of FITC-dextran after stimulation with ETEC K88. The TEER value and the permeability of all treatments were normalized to control. Data values are expressed as mean ± SEM (n = 3). Values with different letters indicate significant difference (P < 0.05).

Compared with ETEC K88, ETEC K88+tryptophan increased the protein concentrations of occludin, ZO-1, claudin-1, and CaSR, but this effect was inhibited by NPS2143 (*P* < 0.05, **Figures 3A–D**). Tryptophan+ETEC K88+NPS2143 significantly reduced the protein concentrations of occludin, ZO-1, claudin-1, and CaSR compared with tryptophan+ETEC K88 (*P* < 0.05, **Figures 3A–D**). Treatment with 0.7 mM tryptophan significantly increased the TEER and significantly decreased the permeability of FITC-dextran in IPEC-J2 cell monolayers compared with untreated cells (*P* < 0.05, **Figures 4A, B****)**. Compared with the control group, treatment with ETEC K88 significantly decreased the TEER and increased the permeability of FITC-dextran in IPEC-J2 cells (*P* < 0.05, **Figures 4A, B****)**. Moreover, treatment with 0.7 mM tryptophan+ETEC K88 significantly decreased the TEER and increased the permeability of FITC-dextran compared with 0.7 mM tryptophan alone (*P* < 0.05, **Figures 4A, B****)**. In the tryptophan+ETEC K88+NPS2143 group, the TEER was significantly reduced, and the permeability of FITC-dextran was significantly increased compared with the tryptophan+ETEC K88 group (*P* < 0.05, **Figures 4A, B****)**.

**Figure 5:**
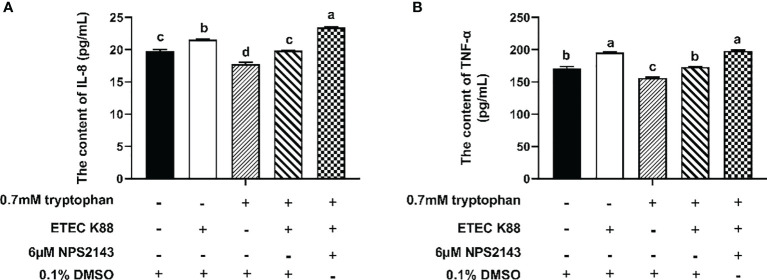
Effect of NPS2143 and tryptophan (0.7mM) on the contents of IL-8 and TNF-α in ETEC K88-stimulated IPEC-J2 cells. IPEC-J2 cells were treated as described in **Figure 3**. **(A)** Effect of NPS2143 and tryptophan on the IL-8 contents in ETEC K88-stimulated IPEC-J2 cells. **(B)** Effect of NPS2143 and tryptophan on the TNF-α contents in ETEC K88-stimulated IPEC-J2 cells. Data values are expressed as mean ± SEM (n = 3). Values with different letters indicate significant difference (P < 0.05).

As shown in **Figures 5A, B**, ETEC K88 significantly decreased the contents of IL-8 and TNF-α in IPEC-J2 cells compared with the control group. Compared with the control group, cells treated with tryptophan had decreased IL-8 and TNF-α contents. Compared with cells treated with tryptophan alone, cells treated with tryptophan+ETEC K88 showed increased contents of IL-8 and TNF-α. However, the incubation of IPEC-J2 cells with NPS2143 reversed the effects of tryptophan on IL-8 and TNF-α contents.

### CaSR Is Required for Tryptophan-Induced Rac1/PLC-γ1 Signaling Activation

**Figure 6:**
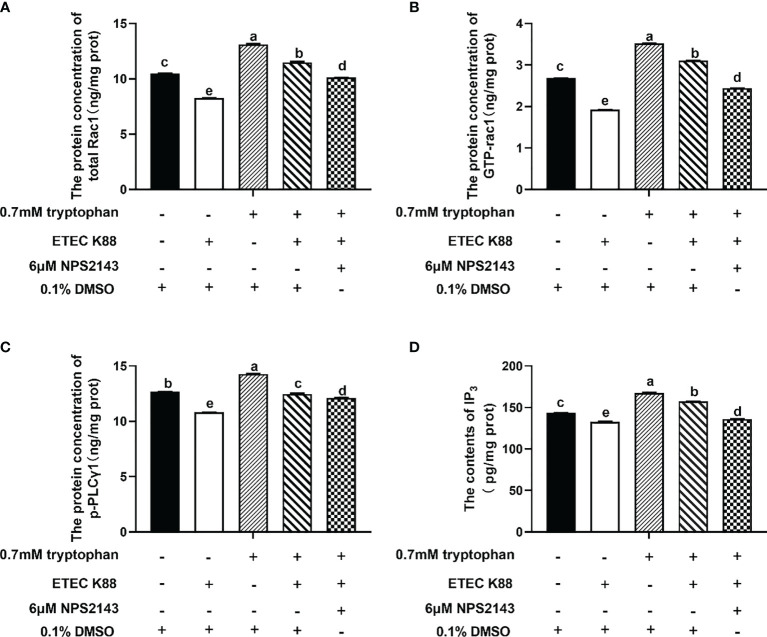
Effect of tryptophan (0.7mM) and NPS2143 on the protein concentration of total Rac1, GTP-rac1, p-PLC-γ1 and contents of IP_3_ in ETEC K88-stimulated IPEC-J2 cells. IPEC-J2 cells were treated as described in **Figure 3**. **(A)** The protein concentration of total Rac1, **(B)** GTP-rac1, **(C)** p-PLC-γ1 and **(D)** the contents of IP_3_ were determined by ELISA kit. Data values are expressed as mean ± SEM from four independent experiments (n = 4). Values with different letters indicate significant difference (P < 0.05).

**Figure 7:**
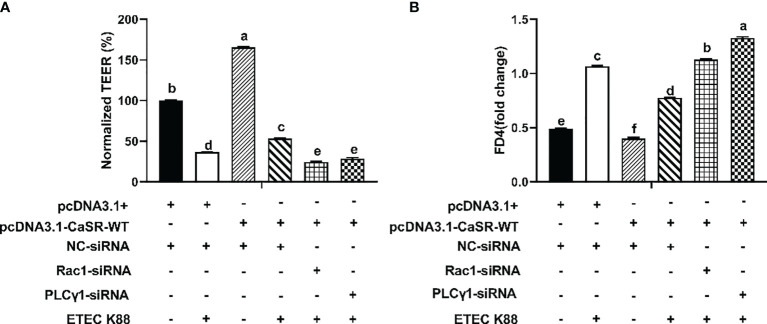
Effect of pcDNA3.1-p(CaSR), Rac1-siRNA, PLC-γ1-siRNA and ETEC-K88 on transepithelial electrical resistance and permeability in IPEC-J2 cells. The IPEC-J2 cells were transfected with pcDNA3.1-p(CaSR), NC-siRNA, Rac1siRNA or PLC-γ1 siRNA for 48 h, followed by ETEC K88 (2% fetal bovine serum DMEM/F12 medium without antibiotic, 1×10^8^ CFU/mL) for 2h. In control and ETEC K88 groups, cells were transfected with NC-siRNA and pcDNA3.1(+). In pcDNA3.1-CaSR-WT+ ETEC K88 groups, cells were transfected with NC-siRNA. **(A)** Effect of ETEC K88, pcDNA3.1-p(CaSR), Rac1siRNA and PLC-γ1 siRNA on the transepithelial electrical resistance value after stimulation with ETEC K88. **(B)** Effect of ETEC K88, pcDNA3.1-p(CaSR), Rac1-siRNA and PLC-γ1-siRNA on the permeability of FITC-dextran after stimulation with ETEC K88. The TEER value and the permeability of FITC-dextran of all treatments were normalized to control. Data values are expressed as mean ± SEM (n = 3). Values with different letters indicate significant difference (P < 0.05).

The protein concentrations of total Rac1, Rho family member 1 of porcine GTP-binding protein (GTP-rac1), and phosphorylated phospholipase Cγ1 (p-PLC-γ1) and the contents of inositol triphosphate (IP_3_) were increased by 0.7 mM tryptophan compared with the control group (*P* < 0.05, **Figures 6A–D**). Compared with the control group, ETEC K88 decreased the protein concentrations of total Rac1, GTP-rac1, and p-PLC-γ1 and the contents of IP_3_ (*P* < 0.05, **Figures 6A–D**). Compared with tryptophan+ETEC K88, NPS2143+tryptophan+ETEC K88 inhibited the increase in protein concentrations of total Rac1, GTP-rac1, and p-PLC-γ1 and IP_3_ contents induced by tryptophan, indicating that the CaSR inhibitor attenuated tryptophan-induced Rac1/PLC-γ1 signaling activation (*P* < 0.05, **Figures 6A–D**). Compared with pcDNA3.1(+), pcDNA3.1(+)-CaSR-WT significantly increased CaSR mRNA expression (*P* < 0.05, **Figure S2**). Moreover, compared with the control group, ETEC K88+NC-siRNA+pcDNA3.1(+) treatment significantly reduced the TEER value and enhanced the permeability of FITC-dextran in IPEC-J2 cells. However, pcDNA3.1-CaSR-WT+NC-siRNA treatment significantly increased the TEER value and decreased the permeability of FITC-dextran (*P* < 0.05, **Figures 7A, B****)**. Compared with ETEC K88-treated cells, pcDNA3.1-CaSR-WT increased the TEER value and reduced the permeability of FITC-dextran in ETEC K88-treated cells (*P* < 0.05, **Figures 7A, B****)**. Compared with pcDNA3.1-CaSR-WT+NC-siRNA+ETEC K88, pcDNA3.1-CaSR-WT+Rac1-siRNA+ETEC K88 and pcDNA3.1-CaSR-WT+PLC-γ1-siRNA+ETEC K88 decreased the TEER value and enhanced the permeability of FITC-dextran in IPEC-J2 cells (*P* < 0.05, **Figures 7A, B****)**.

## Discussion

Different cytokines can modify the junctional complex. The proinflammatory roles of TNF-α and IL-8 were linked with ETEC and increased intestinal permeability (38). Thus, TNF-α and IL-8 parameters were selected in this study. We found that ETEC K88 enhanced the contents of TNF-α and IL-8, which is in agreement with a previous article that ETEC K88 induced intestinal proinflammatory response in pigs (8). The TEER and flux of FITC-dextran indirectly reflect the TJs of intestinal epithelial cells and the paracellular permeability of the intestinal epithelium, respectively. Consistent with the above-mentioned finding, ETEC K88 significantly decreased the TEER values and increased the permeability of FITC-dextran in ETEC K88-challenged IPEC-J2 cells, which suggests that the cell damage model was successfully constructed. In this study, tryptophan significantly decreased the contents of TNF-α and IL-8 in ETEC K88-challenged IPEC-J2 cells, suggesting that tryptophan can attenuate ETEC K88-induced proinflammatory response. This finding is in line with that of a previous study, which showed that tryptophan reduced the gene expression of IL-8 and IL-1β in the gut (21). Proinflammatory cytokines have been related to pathogen-induced alteration of TJ proteins (39). Here, tryptophan (0.7 mM) increased the protein concentrations of occludin, ZO-1, and claudin-1 in ETEC K88-challenged and non-challenged IPEC-J2 cells. These findings were consistent with previous studies on pigs (40), Caco-2 cells, and IPEC-1 cells (18, 19). The current study also demonstrated that tryptophan (0.3 and 0.7 mM) significantly increased the TEER values and decreased the permeability of FITC-dextran in ETEC K88-challenged IPEC-J2 cells. Taken together, our results suggested that tryptophan can improve intestinal barrier integrity and decrease proinflammatory response.

The regulation of intestinal barrier integrity and proinflammatory response is complex, involving numerous intracellular molecular signaling and kinases, such as CaSR, PLC signaling, and RHO kinase. These molecules regulate TJ protein expression, TJ assembly, and redistribution by phosphorylation (41–44). CaSR signaling regulates the TEER in the intestine of mice, TJ protein expression, and proinflammatory immune response (21, 26). We found that NPS2143 reversed the enhancement effect of tryptophan on the protein concentrations of ZO-1, occludin, claudin-1, and CaSR and the TEER and decrease of permeability and IL-8 and TNF-α contents. The overexpression of pcDNA3.1-p(CaSR) markedly increased the TEER and decreased the permeability of FITC-dextran. Taken together, these results suggested that tryptophan protects intestinal epithelial barrier integrity and alleviates intestinal inflammation though CaSR signaling. The Rho family of small guanosine triphosphatases, such as Rho, Cdc42, and Rac1, has been reported to regulate the composition and function of TJs (45–48). The PLC-dependent pathway has been demonstrated in the assembly of TJs in MDCK cells (49, 50). In this study, we found that Rac1-siRNA+tryptophan+ETEC K88 or PLC-γ1-siRNA+tryptophan+ETEC K88 reduced the TEER, increased the permeability of FITC-dextran, and enhanced the contents of IL-8 and TNF-α compared with tryptophan+ETEC K88. Collectively, these results suggested that tryptophan can improve intestinal barrier integrity and decrease proinflammatory response at least partly through Rac1/PLC-γ1 signaling in intestinal epithelial cells. The effects of CaSR on the mRNA expression of inflammatory cytokines and intestinal barrier integrity are associated with two downstream effectors Rac1 and PLC-γ1 (28, 30). In the present study, our results showed that tryptophan+ETEC K88+NPS2143 decreased the protein concentrations of GTP-rac1, total Rac1, and p-PLC-γ1 and contents of IP_3_ compared with tryptophan+ETEC K88. Furthermore, we found that the inhibition of Rac1 or PLC-γ1 by Rac1-siRNA and PLC-γ1-siRNA significantly reduced the TEER and increased the permeability of FITC-dextran in cells treated with pcDNA3.1-p(CaSR) and ETEC K88. These results were consistent with those of previous reports, indicating that tryptophan can enhance IPEC-J2 cell migration through the CaSR/Rac1/PLC-γ1 signaling pathway (24). Taken together, these results suggest that CaSR is required for tryptophan-induced activation of Rac1/PLC-γ1 signaling, which increases intestinal epithelial TJ and decreases intestinal epithelial permeability and inflammatory response in IPEC-J2 cells after ETEC K88 challenge.

Collectively, the results suggest that tryptophan can improve intestinal epithelial barrier integrity and decrease inflammatory response through the CaSR/Rac1/PLC-γ1 signaling pathway. This study not only offers new insights into the function of tryptophan, but also indicates the necessity for further investigating the effect of tryptophan on intestinal health *in vivo*.

## Data Availability Statement

The original contributions presented in the study are included in the article/**Supplementary Material**. Further inquiries can be directed to the corresponding author.

## Author Contributions

GL and KG conceived and designed the experiment. KG and GL wrote the paper. GL, KG, and FW performed the research and analyzed the data. GJ, HZ, XC, CW, RZ, GT, JC, JT, and JW contributed to the analysis and manuscript preparation. All authors contributed to the article and approved the submitted version.

## Funding

This project was supported by the Sichuan Science and Technology Program (No. 2020YJ0398) and Specific Research Supporting Program for Discipline Construction in Sichuan Agricultural University (number 03570126).

## Conflict of Interest

The authors declare that the research was conducted in the absence of any commercial or financial relationships that could be construed as a potential conflict of interest.

## Publisher’s Note

All claims expressed in this article are solely those of the authors and do not necessarily represent those of their affiliated organizations, or those of the publisher, the editors and the reviewers. Any product that may be evaluated in this article, or claim that may be made by its manufacturer, is not guaranteed or endorsed by the publisher.
