# Supplementary material for: Tryptophan Ameliorates Barrier Integrity and Alleviates the Inflammatory Response to Enterotoxigenic Escherichia coli K88 Through the CaSR/Rac1/PLC-γ1 Signaling Pathway in Porcine Intestinal Epithelial Cells
Source: Front Immunol. 2021 Oct 21;12:748497. doi: 10.3389/fimmu.2021.748497 (PMC8566706; doi:10.3389/fimmu.2021.748497)
Supplement: Supplementary file 1 [file DataSheet_1.docx]

**Tryptophan Ameliorates Barrier Integrity and Alleviates the Inflammatory Response to Enterotoxigenic *Escherichia coli* K88 through the CaSR/Rac1/PLC-γ1 Signaling Pathway in Porcine Intestinal Epithelial Cells**

**Guangmang Liu^1,2,3*^, Ke Gu^1,2,3^, Fang Wang^1,2,3^, Gang Jia^1,2,3^, Hua Zhao^1,2,3^, Xiaoling Chen^1,2,3^, Caimei Wu****^1,2,3^, Ruinan Zhang^1,2,3^, Gang Tian****^1,2,3^, Jingyi Cai^1,2,3^, Jiayong Tang^1,2,3^, Jing Wang^4^**

^1^ Institute of Animal Nutrition, Sichuan Agricultural University, Chengdu, Sichuan, China, ^2^ Key Laboratory for Animal Disease-Resistance Nutrition, Ministry of Education, Chengdu, Sichuan, China, ^3^ Key laboratory of Animal Disease-resistant Nutrition and Feed, Ministry of Agriculture and Rural Affairs, Chengdu, Sichuan, China, ^4^ Maize Research Institute, Sichuan Agricultural University, Chengdu, Sichuan, China

**TABLE S1 |** Sequence of primers list used for real time PCR assay.

| Gene names | Primer | Sequence | GenBank accession no | Product size(bp) |
| --- | --- | --- | --- | --- |
| CaSR | Forward  Reverse | 5'-TTCAAGTTACCGCAACCATGAG-3'  5'-CCCTCGTGGCAGGTGATG-3' | GU990706.1 | 60 |
| Rac1 | Forward  Reverse | 5'-GCCCTGCATCTTTTGAAAATGT-3'  5'-GGACAATGGTGTCGCACTTCT-3' | NM_001243585.1 | 61 |
| PLC-γ1 | Forward  Reverse | 5'-TGCCGTCAAAGCGCTCTT-3'  5'-TTCTGGATGATGGCGCTCTT-3' | XM_005672938.2 | 78 |
| β-actin | Forward  Reverse | 5'-TGCGGGACATCAAGGAGAA-3'  5'-GCCATCTCCTGCTCGAAGTC-3' | DQ452569.1 | 58 |

**
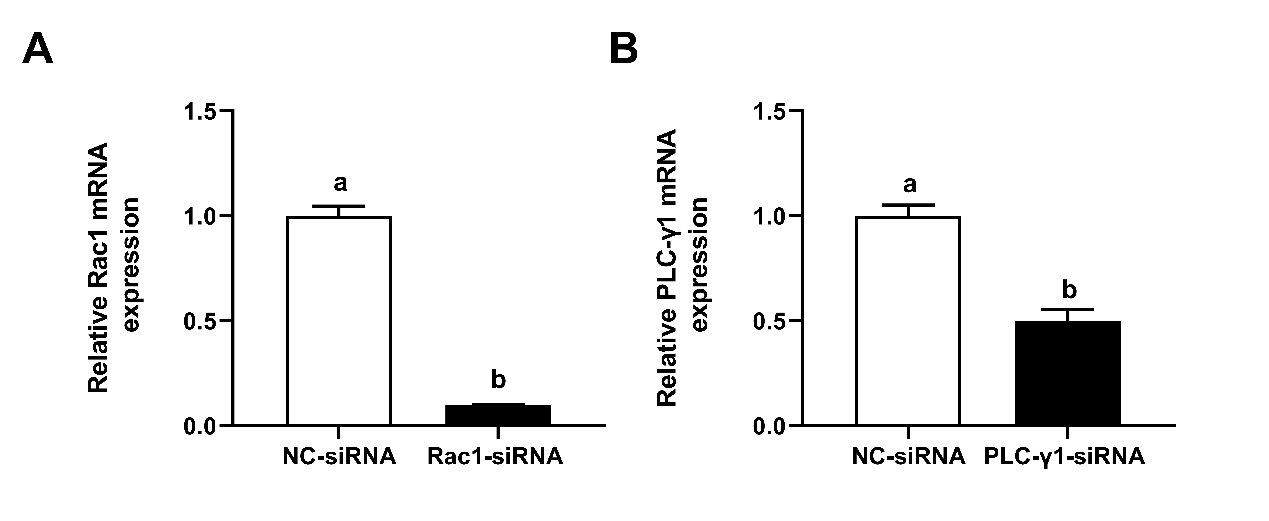
**

FIGURE S1 | Effect of Rac1-siRNA and PLC-γ1-siRNA on Rac1 and PLC-γ1 mRNA expression. The IPEC-J2 cells were transfected with 50nM Rac1-siRNA, PLC-γ1-siRNA and NC-siRNA for 48h. Data values are expressed as mean ± SEM (n = 6). Values with different letters indicate significant difference (P < 0.05).


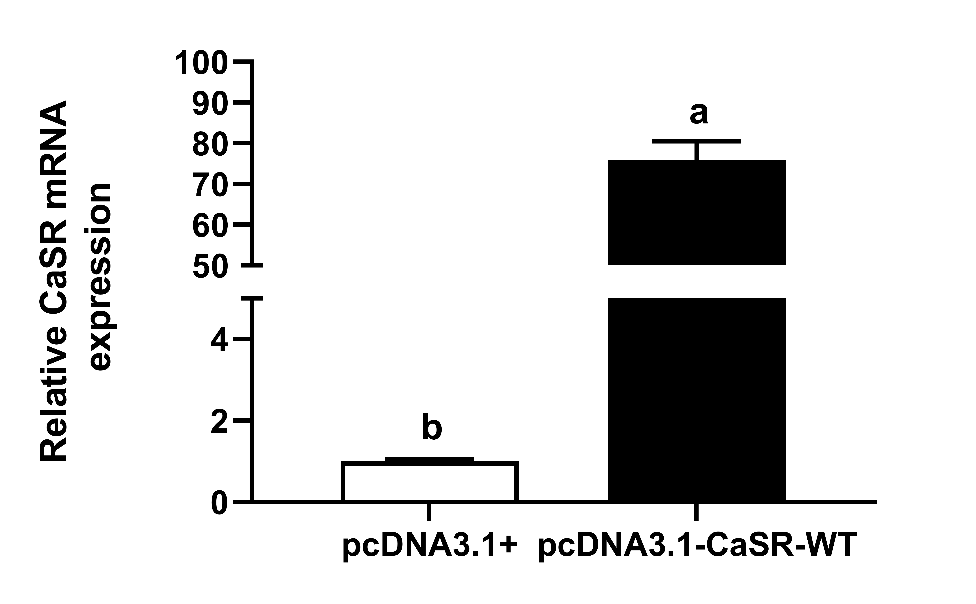


FIGURE S2 | Effect of pcDNA3.1-p(CaSR) on CaSR mRNA expression. The IPEC-J2 cells were transfected with 1.25μg/ml pcDNA3.1-p(CaSR) for 48h. Data values are expressed as mean ± SEM (n = 6). Values with different letters indicate significant difference (P < 0.05).
